# AICAR inhibits ceramide biosynthesis in skeletal muscle

**DOI:** 10.1186/1758-5996-4-45

**Published:** 2012-11-07

**Authors:** Katherine A Erickson, Melissa E Smith, Tamil S Anthonymuthu, Michael J Evanson, Eric S Brassfield, Aimee E Hodson, M Andrew Bressler, Braden J Tucker, Mikayla O Thatcher, John T Prince, Chad R Hancock, Benjamin T Bikman

**Affiliations:** 1 Physiology and Developmental Biology, Brigham Young University, 593 WIDB, Provo, UT 84602, USA; 2 Chemistry and Biochemistry, Brigham Young University, Provo, UT 84602, USA; 3 Nutrition, Dietetics, and Food Science, Brigham Young University, Provo, UT 84602, USA

**Keywords:** AICAR, Ceramide, AMPK, Obesity, Metabolic syndrome

## Abstract

**Background:**

The worldwide prevalence of obesity has lead to increased efforts to find therapies to treat obesity-related pathologies. Ceramide is a well-established mediator of several health problems that arise from adipose tissue expansion. The purpose of this study was to determine whether AICAR, an AMPK-activating drug, selectively reduces skeletal muscle ceramide synthesis.

**Methods:**

Murine myotubes and rats were challenged with palmitate and high-fat diet, respectively, to induce ceramide accrual, in the absence or presence of AICAR. Transcript levels of the rate-limiting enzyme in ceramide biosynthesis, serine palmitoyltransferase 2 (SPT2) were measured, in addition to lipid analysis. Student’s *t*-test and ANOVA were used to assess the association between outcomes and groups.

**Results:**

Palmitate alone induced an increase in serine palmitoyltransferase 2 (SPT2) expression and an elevation of ceramide levels in myotubes. Co-incubation with palmitate and AICAR prevented both effects. However, ceramide and SPT2 increased with the addition of compound C, an AMPK inhibitor. In rats fed a high-fat diet (HFD), soleus SPT2 expression increased compared with normal chow-fed littermates. Moreover, rats on HFD that received daily AICAR injections had lower SPT2 levels and reduced muscle ceramide content compared with those on HFD only.

**Conclusions:**

These results suggest that AICAR reduces ceramide synthesis by targeting SPT2 transcription, likely via AMPK activation as AMPK inhibition prevented the AICAR-induced improvements. Given the role of skeletal muscle ceramide in insulin resistance, it is tempting to speculate that interventions that activate AMPK may lead to long-term ceramide reduction and improved metabolic function.

## Background

Obesity predisposes individuals to a host of health complications, including insulin resistance, hypertension, and dyslipidemia, which characterize the metabolic syndrome [1]. Due to the massive and sustained increase in obesity and the prevalence of the metabolic syndrome worldwide in recent decades [2], extensive efforts have been devoted to better understand the cellular events that mediate obesity-related pathologies.

Long known to regulate apoptotic signals [3], the biologically active sphingolipid ceramide is more recently known to mediate several of the deleterious conditions associated with obesity, such as insulin resistance, hepatic steatosis, and cardiovascular complications [4-6]. A variety of stimuli have been found to induce ceramide biosynthesis in rodents, including high-fat diet [7-9] and inflammatory agonists [8,10,11]. Despite the wealth of research establishing the efficacy of ceramide inhibition in improving obesity-related morbidities [12], no current prescribed pharmacological therapies are known to directly inhibit ceramide biosynthesis. A potentially useful focus in exploring treatments to prevent excessive ceramide accumulation, and block ceramide-mediated pathologies related to obesity, is to identify anti-ceramide effects of currently used drugs [7]. A promising possibility is to explore the numerous drugs prescribed to treat obesity-related diseases that are known to regulate AMP-activated protein kinase (AMPK).

Research in recent decades has elucidated the role of the prototypical metabolic regulator AMPK in ameliorating deleterious conditions associated with obesity. Indeed, AMPK-activating drugs have been shown to improve insulin resistance [13,14], cardiovascular complications [15,16], and hepatic steatosis [17], which may explain their widespread use. Interestingly, many of the benefits attributed to AMPK activation are similar to those seen with ceramide inhibition [4,6,18].

The metabolic functions of AMPK are increasingly clear—increased glucose uptake, increased fatty acid uptake and oxidation, and the inhibition of anabolic processes (e.g., lipogenesis) [13]. We have previously shown that AMPK inhibits inflammatory activity by blocking the NF-κB pathway [19], which, in light of our recent observation that ceramide biosynthesis requires inflammatory signals [8], suggests a potential ceramide-specific effect of AMPK activation. Given the general oxidative effect of AMPK on fatty acids, we sought to test the hypothesis that AMPK inhibits ceramide biosynthesis in muscle. Positive results will reveal a novel mechanism to partly explain the beneficial effects of AMPK-activating drugs in treating several obesity-related pathologies.

## Methods

### Cell culture

Cells were maintained in DMEM + 10% fetal bovine serum (Invitrogen). For differentiation into myotubes, C2C12 myoblasts were grown to confluency and the medium was replaced with DMEM + 10% horse serum (Invitrogen). Myotubes were used for experiments on day 4 of differentiation. For lipid treatment, palmitic acid (Sigma P5585) was dissolved in ethanol and diluted to desired concentration in DMEM. Palmitate solutions were then conjugated to 2% (w/v) BSA (Sigma A9576). Cells were monitored for toxicity to the high palmitic acid concentration (0.75 mM) and no difference was observed between sample protein content. 5-amino-1-β-D-ribofuranosyl-imidazole-4-carboxamide (AICAR) (Sigma A9978) was reconstituted in DMEM. Compound C (Sigma P5499) was reconstituted in DMSO.

### Animal study

Male Wistar rats were kept in a temperature-controlled room with a 12:12-h light–dark cycle. Rats were fed rodent laboratory chow (8604 Harlan Teklad Rodent Diet) and water ad libitum. Treatments lasted 6 wk. During this time, rats were randomly sorted into one of four groups: no intervention (control diet; 8604 Harlan Teklad) (n=9), daily AICAR injections (n=9), high-fat diet (HFD) (n=11), or HFD with daily AICAR injections (n=10). The HFD used in this study was composed of the following (g/kg of food): 116.6 g olive oil, 232.7 g flax seed oil, 87.2 g sugar, 174.6 starch, 226.6 g casein, 4.5 g methionine, 30.7 g gelatin, 51.2 g wheat bran, 22.5 g vitamin mix (Harlan Teklad, AIN76A), 52.2. g mineral mix (Harlan Teklad, AIN76), 1.4 g choline chloride. The HFD was 60% fat (40% flax seed, 20% olive oil). AICAR injections (dissolved in 0.9% NaCl) were administered subcutaneously at a dose of 0.5 mg/g body weight each morning. To reduce confounding factors, rats in the control and HFD groups were similarly handled each morning and injected with similar volumes of saline alone. All experimental procedures involving animals were approved by and performed in compliance with the Institutional Animal Care and Use Committee of Brigham Young University.

### Lipid analysis

For isolation of lipids, cell and tissue pellets were re-suspended in 900 μl ice-cold chloroform:methanol (1:2) and incubated for 15 min on ice then briefly vortexed. A portion of the suspension (prior to pelleting) was used for protein measurement (Thermo Scientific). Separation of aqueous and organic phases required addition of 400 μl of ice-cold water and 300 μl of ice-cold chloroform. The organic phase was collected into a fresh vial and lipids were dried under a gentle nitrogen stream. An Agilent high performance liquid chromatography (HPLC) system coupled with an Applied Biosystems Triple Quadrupole/Ion Trap mass spectrometer (3200 Qtrap) was used for quantification of individual phospholipids. Multiple reaction monitoring (MRM) transitions were set up for quantitative analysis of various polar lipids [20,21]. Levels of individual lipids were quantified using spiked internal standards, including dimyristoyl phosphatidylcholine (28:0-PC), C17-ceramide, C8-glucosylceramide, which were obtained from Avanti Polar Lipids (Alabaster, AL, USA). Neutral lipids were analyzed using a sensitivity HPLC/ESI/MRM method, modified from a previous method [22]. TAG was calculated as relative contents to the spiked d5-TAG 48:0 internal standard (CDN isotopes), while DAG was quantified using 4ME 16:0 Diether DG (Avanti) as an internal standard.

### Quantitative real-time PCR

Total RNA was extracted and purified from tissues using TRIzol (Invitrogen) according to the manufacturer's recommendations. cDNA was synthesized from mRNA by reverse transcription using a commercial cDNA synthesis kit with oligo(dT) primers (iScript Select cDNA Synthesis; Bio-rad). Quantitative real-time PCR was performed by following the instructions accompanying the Qiagen Quantifast SYBER Green PCR kit and using a Qiagen QIAcube. A sample containing no cDNA was used as a non-template control to verify the absence of primer dimers. β-actin reactions were performed side by side with every sample analyzed. Changes in mRNA level of each gene for each treatment were normalized to that of the β-actin control mRNA according to Pfaffle [23]. Sequences are included in Table 1.

**Table 1:** Sequences for PCR primers of indicated genes of interest

**Gene**	**Sequences**
	5’-TGGCATTGTTACCAACTGGG
β-actin	5’-GGGTCATCTTTTCACGGTTG
	5’-TACTCAGAGACCTCCAGCTG
SPT1	5’-CACCAGGGATATGCTGTCATC
	5’-GGAGATGCTGAAGCGGAAC
SPT2	5’-GTATGAGCTGCTGACAGGCA
	5’-CTGTTCTACTTGGCCTGTTG
CerS1/Lass1	5’-TCATGCAGGAAGAACACGAG
	5’-TCTTCTCAAAAAGTTCCGAG
CerS2/Lass2	5’-AGTGATGATGAAAACGAATGG
	5’-TGGCTGCTATTAGTCTGATG
CerS3/Lass3	5’-TCAGGATAAAGTAACCCCAG
	5’-TGTCGTTCAGCTTGAGTGAG
CerS4/Lass4	5’-AGCAGGCTTCACAGAATTTC
	5’-CTCCAACGCTCACGAAATTC
CerS5/Lass5	5’-ATGCAGACAGAAGATGAGTG
	5’-GTTCGGAGCATTCAACGCTG
CerS6/Lass6	5’-CTGAGTCGTGAAGACAGAGG
	5'-CACCGGTACCTCGGAGCGGA
Des1	5'-GTTTGGGATTGATGAACAGGGGT
Glucosylceramide	5’-GCTCAGTACATTGCTGAAGA
Synthase	5’-AGTACGAACCAGAGTTTTGC

### Protein analysis

Gastrocnemius was extracted from euthanized mice and flash frozen then stored at −80. Tissues were later lysed and protein content was determined using a BCA protein assay (Pierce, Rockford, IL, USA) and sample volumes were adjusted so that precisely 50 μg of protein was loaded into each lane. After addition of sample buffer, samples were resolved by SDS-PAGE, transferred to nitrocellulose, and immunoblotted with anti-SPT2 antibody (Abcam, Cambridge, MA, USA) using methods described previously [24]. After incubation with primary antibody, blots were incubated with a horseradish peroxidase-conjugated secondary antibody. Horseradish peroxidase activity was assessed with ECL solution (Thermo Scientific, Rockford, IL, USA) and exposed to film.

### Statistics

Data are presented as the mean ± SEM. Data were compared by ANOVA with Tukey’s post-hoc analysis (Graphpad Prism; La Jolla, CA). Significance was set at *P*<0.05.

## Results

### AICAR selectively blocks sphingolipid accumulation in muscle cells

**Figure 1:**
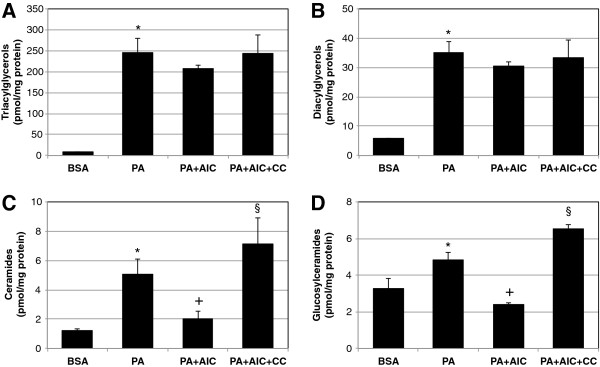
**Palmitate-induced sphingolipid accumulation is prevented with AICAR. **C2C12 myotubes were incubated with palmitate (PA) for 16 h at 0.75 mM. PA treatment increased all measured lipids. However, while ceramides (**C**) and glucosylceramides (**D**) were reduced with AICAR inclusion (2 mM), triacylglycerols (**A**) and diacylglycerols (**B**) were not significantly affected. Similarly, the inclusion of Compound C (20 μM) with AICAR (PA+AIC+CC) increased lipid levels over PA+AIC in ceramides (**C**) and glucosylceramides (**D**). *p<0.05 for PA vs. BSA. +*p*<0.05 for PA+AIC vs PA. §*p*<0.05 for PA+AIC+CC vs. PA+AIC (n=5).

AMPK activation is known to increase rates of mitochondrial fatty acid oxidation [25]. Based on these observations, we anticipated a general reduction in accumulated intracellular lipids when cells were treated with palmitate and AICAR when compared with palmitate treatment alone. AICAR is a well-established AMPK activator. We found that levels of the glycerolipids triacylglycerol (TAG) and diacylglycerol (DAG) (Figure 1A and B, respectively) were significantly increased in C2C12 myotubes in response to prolonged palmitate treatment. AICAR addition to palmitate incubations had no significant effect on TAG and DAG levels. Similar to these glycerolipids, palmitate induced a robust increase in ceramides and glucosylceramides (Figure 1C and D, respectively), which have both been implicated in mediating lipid-induced insulin resistance [26,27]. However, whereas AICAR treatment had little effect of TAG and DAG, AICAR treatment blunted ceramide and glucosylceramides accrual to such a degree that levels returned to vehicle-treated conditions (Figure 1C and D). Despite its well-established ability to activate AMPK, AICAR may have off-target effects. To more confidently establish the importance of AMPK in mediating the sphingolipid-specific effects of AICAR, we included compound C, a specific AMPK inhibitor. The addition of compound C to palmitate and AICAR treatments completely reversed the protective effect of AICAR with ceramides (Figure 1C) and glucosylceramides (Figure 1D).

### AICAR prevents lipid-induced transcription of SPT2 in muscle cells

**Figure 2:**
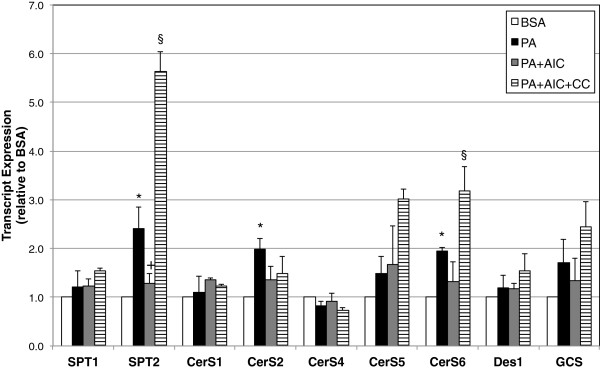
**Palmitate increases transcription of multiple enzymes of de novo sphingolipid synthesis.** C2C12 myotubes were treated with palmitate (PA; 16 h, 0.75 mM) in the absence (PA) or presence of AICAR (PA+AIC; 2 mM). Levels of serinepalmitoyl transferase 2 (SPT2) and ceramide synthase 2 and 6 (CerS 2 and 6) were elevated with PA, but only SPT2 was significantly affected by inclusion of AICAR. Moreover, addition of Compound C (20 μM) caused a robust increase in SPT2 and, to a lesser degree, CerS6 transcript levels. SPT1-2: serine palmitoyltransferase 1–2; CerS 1–6: ceramide synthase 1–6; Des1: dihydroceramide desaturase 1; GCS: glucosylceramide synthase. *p<0.05 for PA vs BSA. +*p*<0.05 for PA+AIC vs PA. §*p*<0.05 for PA+AIC+CC vs. PA+AIC (n=4).

Given our findings that AICAR inhibited the synthesis of ceramide and its downstream metabolite, glucosylceramide, we sought to determine whether AICAR alters expression of enzymes involved in de novo sphingolipid biosynthesis. In support of previous findings [8], palmitate elicited a significant increase in transcript expression of multiple enzymes, including ceramide synthase 6 (CerS6), CerS2, and, finally, serine palmitoyltransferase 2 (SPT2) (Figure 2). The observations regarding SPT2 are doubly noteworthy—SPT2 acts as the rate-limiting enzyme in de novo sphingolipid synthesis and it was the only enzyme whose transcript level was significantly blunted with inclusion of AICAR into the palmitate-enriched media (Figure 2). Interestingly, addition of the AMPK-inhibitor compound C elicited a pronounced increase in SPT2 and CerS6 transcript levels.

### AICAR prevents high-fat diet-induced SPT2 expression in vivo

**Figure 3:**
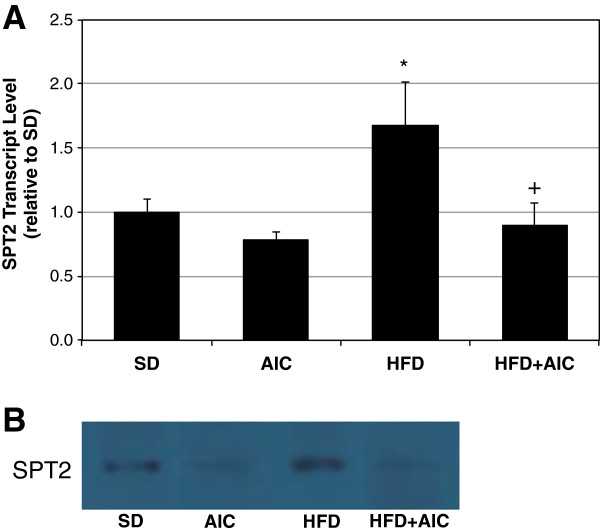
**AICAR inhibits SPT2 induction. **Male Wistar rats were fed a high-fat diet (HFD) and received daily injections of vehicle (saline) or AICAR (AIC; 0.5 mg/g body weight) for 6 wk. Daily AICAR injections prevented the high-fat diet-induced increase in SPT2 transcription (**A**) and resulted in decreased SPT2 protein levels (**B**). *p<0.05 for HFD vs Chow. +*p*<0.05 for HFD+AICAR vs HFD (n=9-11).

**Figure 4:**
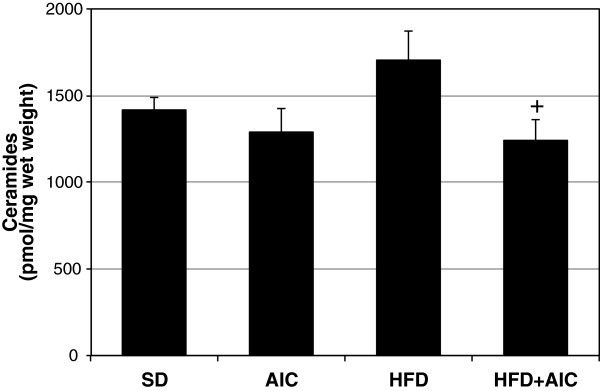
**AICAR prevents high-fat diet-induced ceramide accumulation in muscle. **Male Wistar rats were fed a high-fat diet (HFD) and received daily injections of vehicle (saline) or AICAR (AIC; 0.5 mg/g body weight) for 6 wk. HFD alone did not significantly increase ceramides compared with the Chow-fed group, but HFD+AIC resulted in a significant reduction in ceramides compared with HFD alone. +*p*<0.05 for HFD+AICAR vs HFD (n=9-11).

Having established an anti-ceramide effect of AICAR in cultured muscle cells, we next explored this relationship in whole muscle. Male Wistar rats were fed a normal chow or high-fat diet for 6 wk. During this period, animals further received daily injections of AICAR (0.5 mg/g BW) or vehicle (saline). Given our observations that SPT2 was affected by both the lipid-laden and AICAR conditions in cells, we analyzed SPT2 expression in the muscles of rats in the control, HFD, and HFD+AICAR conditions. We found that SPT2 transcript levels increased by roughly 60% in the muscle of animals fed a HFD when compared with those receiving normal laboratory chow (Figure 3A). Importantly, prolonged AICAR injections prevented this effect, with SPT2 levels falling slightly lower than control conditions (Figure 3A). These findings were further supported by similar results with SPT2 protein levels (Figure 3B). However, the increased SPT2 transcription was insufficient to significantly increase muscle ceramide levels in rats on HFD compared with normal chow, which only tended to increase (*p*=0.068) (Figure 4). Nevertheless, ceramide content from muscles of rats fed a HFD while receiving AICAR injections was significantly lower than those on HFD alone (Figure 4).

## Discussion

The purpose of this study was to elucidate the targeted effect of AICAR on inhibiting de novo ceramide biosynthesis in skeletal muscle. Our data suggest that AICAR treatment, which is known to potently activate AMPK, inhibits ceramide accumulation in lipid-laden conditions by inhibiting lipid-induced expression of the rate-limiting enzyme SPT2. These findings were particularly robust in murine myotubes, which showed a dramatic increase in SPT2 expression and ceramides with palmitate exposure and a complete reversal of both trends with AICAR inclusion. Additionally, and suggestive of the importance of AMPK, the addition of the AMPK inhibitor compound C increased transcript levels of SPT2 (Figure 2) and both sphingolipids (Figure 1C and D). Less dramatic were the data from muscle from HFD-fed and AICAR-treated rats. While SPT2 expression was increased in muscle from HFD-fed rats compared with those receiving normal chow and decreased in the muscle of rats receiving AICAR injection in conjunction with HFD, actual ceramides only significantly differed between the HFD and HFD plus AICAR groups, nonetheless suggesting an *in vivo* effect of AICAR on ceramide inhibition (Figure 4).

A likely explanation for the lack of a more robust increase in muscle ceramides in the HFD-fed group is the saturation state of the lipids in the HFD. Previous studies that have shown increased muscle ceramide in HFD-fed models have used a HFD that is largely lard based and is, subsequently, much higher in saturated fats than the HFD used in this study, which was almost completely unsaturated (flax and olive oil). Saturated fat is not only a substrate for ceramide, but it also activates the biosynthetic pathway [8,28], while unsaturated fats, particularly the mono-unsaturated oleic acid, do not [7]. In contrast to the *in vivo* findings, our use of palmitate in the cell culture provided a strong induction of ceramide biosynthesis. While the concentration used is not physiological (0.75 mM), it is widely used as a method to potently activate ceramide biosynthetic pathways.

Our findings of an anti-ceramide effect of AICAR in skeletal muscle corroborate previous observations in non-muscle tissues. Blazquez et al. [29] found that long-term stimulation of AMPK via AICAR treatment in astrocytes inhibited SPT activity and reduced ceramides. Collectively, these data highlight a novel specificity of AMPK activation to target ceramide synthesis in various cell types. Further, a possible mechanism for AMPK-induced inhibition of ceramide biosynthesis is the anti-inflammatory effects of AMPK. We recently found that ceramide biosynthesis requires NF-κB activation [8], which is inhibited by AMPK. Cacicedo et al. [30] observed that active AMPK prevents the inflammatory effects of both saturated fatty acids (SFA) and TNFα in endothelial cells. In particular, they found that the SFA- and TNFα-induced 2- to 4-fold increase in NF-κB reporter gene expression was prevented with both AICAR treatment and constitutively active AMPK [30].

Many prominent AMPK-activating drugs like metformin and thiazolidinediones (TZD) possess anti-inflammatory activity [31,32]. Thus, given previous findings, it is not surprising that both metformin and TZD reduce ceramide accumulation [33,34], though whether the activation of AMPK is necessary for the drugs’ anti-inflammatory and anti-ceramide actions is unknown.

Previous studies have tangentially explored the relationship between AMPK and ceramides in skeletal muscle. Holland et al. [35] found that adiponectin, long known to improve insulin sensitivity and activate AMPK [36], potently stimulates ceramide conversion to sphingosine 1-phosphate (S1P), which opposes several of ceramide’s deleterious effects in the cell [37]. Interestingly, S1P induces AMPK activation, which explains the observation by Holland et al. [35] that ceramide activates AMPK in muscle cells. Given our findings that AMPK inhibits ceramide biosynthesis in muscle, these studies collectively suggest a self-regulating mechanism, wherein ceramide is converted to S1P, which activates AMPK and, in turn, inhibits ceramide biosynthesis.

## Conclusions

In conclusion, these experiments reveal that the AMPK activator AICAR reduces expression of SPT2 in skeletal muscle and prevents ceramide biosynthesis in hyperlipidemic conditions. Given the wide range of AMPK-activating drugs currently used to mitigate obesity-induced pathologies, our findings suggest that ceramide inhibition may be an important and novel mechanism that mediates several health benefits derived from AMPK activation.

## Competing interests

The authors declare no competing interests.

## Authors’ contributions

KAE and MES assisted with cell treatments, RT-qPCR, lipid extraction, and manuscript preparation. TSA and JTP performed lipid analysis. MJE, ESB, AEH, MAB, BJT, MOT assisted with RT-qPCR and lipid extraction. CRH managed the animal study and assisted with manuscript preparation. BTB was involved in experimental planning, all experiments, and manuscript preparation. All authors read and approved the final manuscript.

## Authors’ information

Co-first authors: Katherine A. Erickson and Melissa E. Smith.
